# Neuronal control of lipid metabolism by STR‐2 G protein‐coupled receptor promotes longevity in *Caenorhabditis elegans*

**DOI:** 10.1111/acel.13160

**Published:** 2020-05-20

**Authors:** Anubhuti Dixit, Anjali Sandhu, Souvik Modi, Meghana Shashikanth, Sandhya P. Koushika, Jennifer L. Watts, Varsha Singh

**Affiliations:** ^1^ Department of Molecular Reproduction, Development and Genetics Indian Institute of Science Bangalore India; ^2^ Department of Biological Sciences Tata Institute of Fundamental Research Mumbai India; ^3^ School of Molecular Biosciences Washington State University Pullman WA USA; ^4^ Present address: Amity Institute of Neuropsychology and Neurosciences Amity University Noida India

**Keywords:** *C. elegans*, chemosensory GPCR, desaturases, diet, lipid metabolism, longevity, temperature

## Abstract

The G protein‐coupled receptor (GPCR) encoding family of genes constitutes more than 6% of genes in *Caenorhabditis elegans* genome. GPCRs control behavior, innate immunity, chemotaxis, and food search behavior. Here, we show that *C. elegans* longevity is regulated by a chemosensory GPCR STR‐2, expressed in AWC and ASI amphid sensory neurons. STR‐2 function is required at temperatures of 20°C and higher on standard *Escherichia coli* OP50 diet. Under these conditions, this neuronal receptor also controls health span parameters and lipid droplet (LD) homeostasis in the intestine. We show that STR‐2 regulates expression of delta‐9 desaturases, *fat‐5*,* fat‐6* and* fat‐7*, and of diacylglycerol acyltransferase *dgat‐2*. Rescue of the STR‐2 function in either AWC and ASI, or ASI sensory neurons alone, restores expression of *fat‐5*, *dgat‐2* and restores LD stores and longevity. Rescue of stored fat levels of GPCR mutant animals to wild‐type levels, with low concentration of glucose, rescues its lifespan phenotype. In all, we show that neuronal STR‐2 GPCR facilitates control of neutral lipid levels and longevity in *C. elegans*.

## Graphical abstract

Neuronal G protein‐coupled receptor (GPCR), STR‐2, regulates fat storage and lifespan in *Caenorhabditis elegans* on standard *Escherichia coli* OP50 diet, at 20°C or higher. STR‐2 regulates adaptation of *C. elegans* AWC^on^ neuron and ASi neuron to increase in temperature, and it fine tunes metabolic adaptation of worms to increase in temperature.

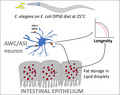

## 1 INTRODUCTION

G protein‐coupled receptors (GPCRs) regulate important biological processes in eukaryotes. They regulate the physiology of organ systems, metabolism, lifespan, and behavior. The divergent structure and ligand binding ability of GPCRs allows them to respond to a large variety of chemical, mechanical, and light stimuli. Apart from sensing external cues, GPCRs play central roles in maintaining metabolic homeostasis. In higher animals, GPCRs such as GPR43, GPR119, and GPR120 regulate metabolic homeostasis in response to ghrelin and lipids (Gong et al., 2014; Talukdar, Olefsky, & Osborn, 2011).

G protein‐coupled receptor repertoire is often larger in lower animals which reside closer to the soil and largely rely on chemosensory cues for perception of food, potential mates, or pathogens. For example, the soil dwelling roundworm *Caenorhabditis elegans* has ~1,500 GPCR encoding genes in its genome (Troemel, Chou, Dwyer, Colbert, & Bargmann, 1995). Earlier, we have shown that two of these GPCRs, NPR‐1 and OCTR‐1, regulate innate immune responses of *C. elegans* to Gram negative bacterium *Pseudomonas aeruginosa* (Styer et al., 2008; Sun, Singh, Kajino‐Sakamoto, & Aballay, 2011). DCAR‐1 GPCR in the *C. elegans* hypodermis regulates immune response to fungal invasion and infection through the skin (Zugasti, 2014). Other GPCRs regulate stimulus‐dependent chemotactic behavior, such as ODR‐10; a GPCR expressed in the olfactory neuron AWA is responsible for chemotaxis to diacetyl, a volatile produced by many bacteria (Troemel et al., 1995).

G protein‐coupled receptors and sensory neurons play important roles in the regulation of lifespan. Or83b odorant receptor GPCR in fruit flies regulates olfaction, lifespan, and caloric restriction response (Libert et al., 2007). Several sensory neurons, including olfactory neurons, in *C. elegans* control longevity (Alcedo & Kenyon, 2004). Olfactory neurons of *C. elegans* play important roles in food search behavior, avoidance of pathogens, and thermosensation. Serotonin regulates food search behavior and lipid metabolism (Srinivasan et al., 2008), while serotonin receptors SER‐1 and SER‐4 antagonistically regulate lifespan (Murakami & Murakami, 2007). These studies suggest a close link between food sensing, metabolism, and lifespan.

In this study, we asked if olfactory GPCRs regulate lifespan in *C. elegans*. We report that chemosensory GPCR STR‐2 is necessary for maintenance of normal lifespan of *C. elegans* on laboratory diet *Escherichia coli* OP50, at growth temperatures of 20°C and above. Under these conditions, STR‐2 in AWC and ASI neurons regulates pharyngeal pumping, body length, and lipid droplet (LD) homeostasis in the intestine. STR‐2 regulates transcription of key lipid metabolism enzymes for lipid desaturation and LD synthesis. Rescue of neutral lipid stores rescues the lifespan phenotype of short‐lived animals. We propose that neuronal STR‐2 GPCR helps in physiological adaptation to diet and temperature by fine tuning lipid metabolism in non‐neuronal tissues.

## 2 RESULTS

### 2.1 Olfactory GPCR STR‐2 positively regulates *C. elegans* lifespan, pharyngeal pumping, and body length in a temperature‐dependent manner

**FIGURE 1:**
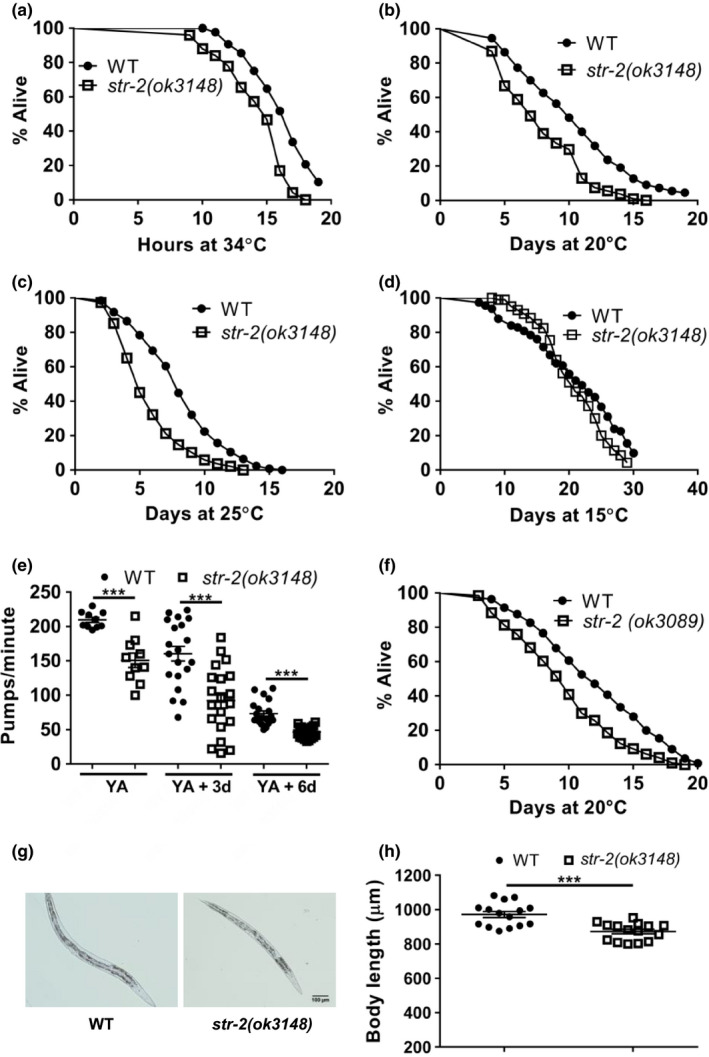
STR‐2 regulates lifespan and health span of *Caenorhabditis elegans* in a temperature‐dependent manner. Kaplan–Meier survival curves of wild‐type (WT) animals along with (a) *str‐2(ok3148)* at 20°C, (b) *str‐2(ok3089)* at 20°C, (c) *str‐2(ok3148)* at 25°C, and (d) *str‐2(ok3148)* at 15°C. (e) Pharyngeal pumping rates as measured by pharyngeal contractions for WT and *str‐2(ok3148)* animals at 25°C. (f) Kaplan–Meier survival curves of WT and *str‐2(ok3148)* animals at 34°C. (g) Bright‐field images of WT and *str‐2(ok3148)* animals. (h) Body length measurements for WT and *str‐2(ok3148)* animals at 25°C. See also Figures S1, S2 and Table S1 for mean lifespan and statistics. Each dot represents data from individual animals. ***p < .001

AWA and AWC neurons are primary neurons known for recognition of bacterial food odors in *C. elegans* (Bargmann, Hartwieg, & Horvitz, 1993). We examined the involvement of the ODR‐10 GPCR, expressed in AWA neurons, SRA‐13 GPCR, expressed in AWA and AWC neurons, and STR‐2 GPCR, expressed in AWC and ASI neurons. Lifespan of *odr‐10(ky32)* and *sra‐13(zh3)* was similar to wild‐type (WT) N2 or WT animals (Figure S1A,B), while *str‐2(ok3089)* animals lived shorter than WT (Figure 1a; Table S2). Another mutation in *str‐2, ok3148* allele*,* also showed a short‐lived phenotype (Figure 1b). From here on, we have used *str‐2(ok3148)* allele for all the experiments. Since the STR‐2 receptor is expressed in both AWC and ASI neurons, which are thermo sensory (Kimata, Sasakura, Ohnishi, Nishio, & Mori, 2012), we tested the lifespan phenotype of *str‐2* animals at temperature lower and higher than 20°C. Surprisingly, we found that *str‐2* animals had WT lifespan at 15°C, while they were short lived at 25°C (Figure 1c,d; Table S2). We would like to note that lifespan of WT animals at 15, 20 and 25°C in some of our assays is on the lower side of reported values for *C. elegans* (see Table S2 for details). To understand whether STR‐2 function is required during larval development, we allowed WT and *str‐2* worms to go through larval development at 25°C and then shifted them to 15°C for lifespan assays. We found that *str‐2* animals had a shorter lifespan than WT animals (Figure S1C) suggesting that STR‐2 function during larval development is crucial for longevity later in adult life. Together, these findings suggested that the STR‐2 receptor is required for longevity at temperatures of 20°C or higher and pointed to a possible role in temperature‐mediated lifespan regulation.

Shortening in the lifespan could results from aging in one organ systems such as the nervous system, reproductive system, musculature or due to decline in stress responses. Indeed, we found that *str‐2* animals showed a significant reduction in pharyngeal pumping in comparison with WT animals at the young adult (YA) stage, 3D adult, and 6D adult (Figure 1e). Body length of worms is associated with feeding in *sma* mutants, defective in TGFβ signaling (Gomez‐Amaro et al., 2015). We observed a significant decrease (around 12%) in body length of *str‐2* animals in comparison with the WT animals (Figure 1g,h). We further asked whether *str‐2* mutation caused change in other health span parameters. The *str‐2* animals showed WT levels of age‐related fluorescent pigment, lipofuscin (Figure S2A,B). *str‐2* animals also had normal brood size at 25°C (Figure S2C) indicating that STR‐2 did not affect reproductive capacity. The *str‐2* animals had WT resistance to oxidative stress (Figure S2D), and to pathogenic bacteria, *P. aeruginosa* and *Enterococcus faecalis* (Figure S2E,F) at 25°C. However, *str‐2* animals were more susceptible to heat stress (Figure 1f). Since *str‐2* animals showed WT longevity at 15°C, we tested whether pharyngeal pumping and body length were regulated by temperature. WT animals grown at 15°C showed slower pumping rate in comparison with the WT animals grown at 25°C (Figure S2G,H) as shown earlier (Chauhan, Orsi, Brown, Pritchard, & Aylott, 2013). Interestingly, we found that the body length and the pharyngeal pumping of *str‐2* animals were comparable to those of WT animals at 15°C (Figure S2G,H), pointing to a function of this GPCR only at higher temperature. These findings suggest that STR‐2 receptor activity controls some health span parameters at higher temperature as well as heat stress resistance.

### 2.2 STR‐2 function in AWC/ASI neuron is sufficient for longevity

**FIGURE 2:**
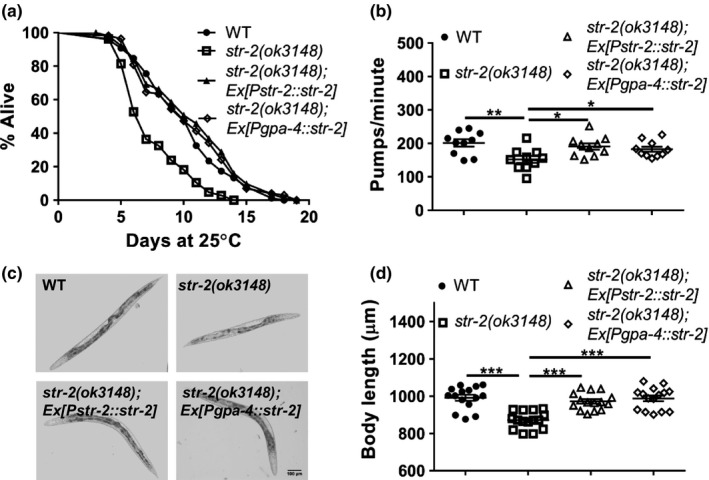
STR‐2 functions in AWC or ASI neurons to regulate lifespan of worms. (a) Kaplan–Meier survival curves for wild‐type (WT), *str‐2(ok3148)* and *str‐2(ok3148)*; Ex[P*str‐2*::*str‐2*] and *str‐2(ok3148);*Ex[P*gpa‐4*::*str‐2*] animals. (b) Pharyngeal pumping rates of WT, *str‐2(ok3148)*, *str‐2(ok3148)*;Ex[P*str‐2*::*str‐2*] and *str‐2(ok3148);*Ex[P*gpa‐4*::*str‐2*] animals. (c,d) Bright‐field images and body length measurements of WT, *str‐2(ok3148)*, *str‐2(ok3148)*;Ex[P*str‐2*::*str‐2*] and *str‐2(ok3148);*Ex[P*gpa‐4*::*str‐2*] animals. Each dot represents data from individual animals. *p < .05, **p < .01, ***p < .001. See also Figure S3 and Table S2 for mean lifespan and statistics

The *str‐2* gene is expressed in one of the two AWC neurons which is termed AWC^on^, while the other neuron is termed AWC^off^. This gene is also expressed weakly in both of the ASI neurons (Troemel, Sagasti, & Bargmann, 1999). For complementation, *str‐2* ORF was expressed in AWC and ASI neurons under *str‐2* promoter or in ASI neurons alone using *gpa‐4* promoter. Both showed rescue of short lifespan phenotype of *str‐2* animals (Figure 2a). Complementation of *str‐2* in AWC + ASI or ASI alone also rescued the pharyngeal pumping defect as well as body length shortening at 25°C (Figure 2b–d). However, over‐expression of *str‐2* in WT animals did not increase longevity (Figure S3) suggesting complex regulation of GPCR activity. Complementation of *str‐2* in AWC and ASE neurons, under *ceh‐36* promoter, led to partial rescue of lifespan phenotype of *str‐2* animals (Figure S3B). Complementation of the gene under an AWC‐specific promoter could not be done because of lack of an AWC^on^ only promoter except for the *str‐2* promoter itself. Taken together, these findings demonstrate that STR‐2 function in AWC^on^ or ASIL/R neuron is sufficient for rescue of lifespan and health span phenotypes.

### 2.3 STR‐2 regulates LD homeostasis and lipid metabolism

**FIGURE 3:**
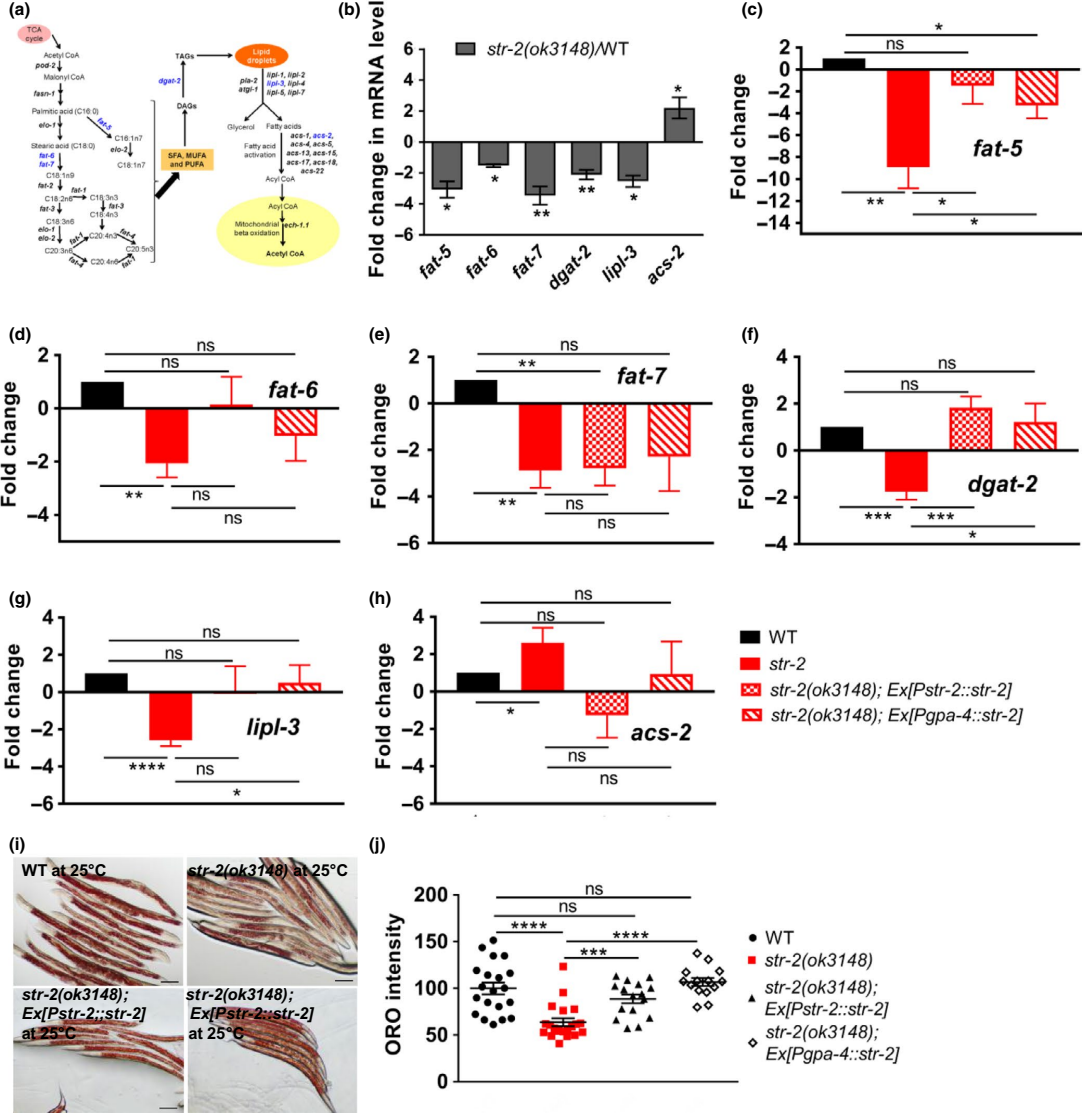
STR‐2 regulates lipid metabolism and lipid droplet (LD) homeostasis. (a) Schematic of lipid metabolism. Genes depicted were used for qPCR analysis. Genes shown in blue color were differentially regulated in our study. (b) qPCR analysis of lipid metabolism genes in wild‐type (WT) and *str‐2(ok3148)* animals at 25°C fed *Escherichia coli* OP50. qPCR analysis of (c) *fat‐5,* (d) *fat‐6,* (e) *fat‐7,* (f) *dgat‐2,* (g) *lipl‐3, and* (h) *acs‐2 in* WT, *str‐2 (ok3148)*, *str‐2(ok3148)*; Ex[P*str‐2*::*str‐2*] and *str‐2(ok3148);*Ex[P*gpa‐4*::*str‐2*] animals. (i) Oil Red O staining of LDs (scale bar 100 µM), and (j) Quantification of LD stores in WT, *str‐2 (ok3148)*, *str‐2* (*ok3148*);*str‐2(ok3148)*; Ex[P*str‐2*::*str‐2*] and *str‐2(ok3148);*Ex[P*gpa‐4*::*str‐2*] animals at 25°C. Each dot represents data from individual animal. *p < .05, **p < .01, ***p < .001, ns = nonsignificant

We sought to determine possible mechanisms by which STR‐2 in just three neurons regulates lifespan at temperatures above 20°C. *C. elegans* can adapt to altered temperature by changing the levels of lipid desaturases (Ma et al., 2015; Savory, Sait, & Hope, 2011). We hypothesized that the STR‐2 receptor fine tunes fatty acid composition for adaption to temperature change. As a first step, we analyzed transcript levels for 31 enzymes involved in lipid synthesis and hydrolysis by quantitative real‐time PCR (qPCR) (Figure 3a,b; Figure S4). We found that majority of them were not significantly altered in *str‐2* animals (Figure S4). However, expression of some key enzymes involved in fatty acid desaturation, LD synthesis, and lipid hydrolysis was altered significantly in *str‐2* animals fed on *E. coli* OP50 at 25°C (Figure 3b). The *str‐2* animals showed significant downregulation of transcript levels of delta‐9 desaturases *fat‐5, fat‐6, and fat‐7,* diacylglycerol acyltransferase *dgat‐2* (rate‐limiting enzyme for triacylglycerol [TAG] synthesis), lipase *lipl‐3,* and upregulation of the transcript for acyl‐CoA synthetase *acs‐2* in comparison with WT animals (Figure 3b). We also tested if the dysregulation of lipid metabolism had any impact on neutral lipids stored in LDs. Based on reduced desaturase and *dgat‐2* expression combined with reduced body length, we predicted that *str‐2* animals would have reduced fat stores. Indeed, we found that *str‐2* animals had 20%–30% fewer Oil Red O stained LDs than WT animals (Figure 3i,j). This phenotype is similar to reduced LD phenotype of *fat‐6, fat‐7* double mutant animals (Brock, Browse, & Watts, 2007), but less substantial. We tested if the deficit in desaturase and *dgat‐2* expression due to *str‐2* mutation continues with age. Interestingly, 3‐day adults—WT as well as *str‐2*—showed more LDs than young animals (Figure S5A) of the same genotype as shown for WT at 20°C (Amrit et al., 2016). Three‐day *str‐2* adults showed LD levels similar to 3‐day WT adults (Figure S5B). Older animals showed increased levels of all desaturases (Figure S5C,D) in both WT and *str‐2* animals suggesting that maintaining levels of monounsaturated fatty acids (MUFA) or total neutral lipid may be important during aging in *C. elegans*. Even in 3‐day adults, transcript levels of desaturases and *dgat‐2* were lower in *str‐2* than in WT animals of the same age suggesting that deficit due to the *str‐2* mutation continues with age.

Complementation of *str‐2* gene in AWC + ASI neurons or ASI alone could partly rescue lipid metabolism changes (Figure 3c–h). Expression of *fat‐5* and *dgat‐2* transcripts was restored in AWC + ASI as well as ASI complemented strain. Importantly, STR‐2 complemented strains also showed restoration of LD stores in the intestine up to WT levels (Figure 3i,j). In all, these experiments suggested that fat storage and MUFA level maintenance at 25°C required neuronal activity of STR‐2 GPCR.

### 2.4 STR‐2 mediates metabolic adaptation to high temperature

**FIGURE 4:**
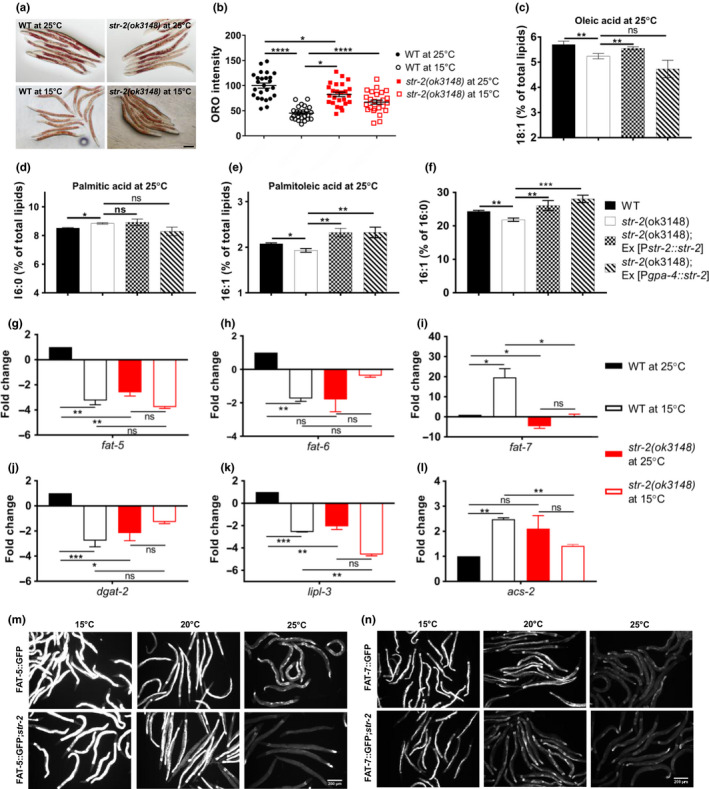
STR‐2 regulates lipid metabolism at high temperature. (a) Lipid stores stained by Oil Red O (scale bar 100 µM) and (b) quantification of lipid stores in wild‐type (WT) and *str‐2(ok3148)* animals grown at 15°C or 25°C. Each dot represents data from individual animal. Bar diagrams showing levels of (c) oleic acid, (d) palmitic acid, (e) palmitoleic acid, (f) ratio of palmitoleic acid to palmitic acid in WT, *str‐2 (ok3148)*, *str‐2* (*ok3148*);*str‐2(ok3148)*; Ex[P*str‐2*::*str‐2*] and *str‐2(ok3148);*Ex[P*gpa‐4*::*str‐2*] animals. (g) qPCR analysis of lipid metabolism genes (g) *fat‐5*, (h) *fat‐6*, (i) *fat‐7*, (j) *dgat‐2*, (k) *lipl‐3*, and (l) *acs‐2* in *str‐2 (ok3148)* and WT animals grown at 25°C or at 15°C. All the fold changes are with respect to WT animals at 25°C. (m) fat‐5::GFP expression in WT and *str‐2(ok3148)* animals at 15, 20, and 25°C. (n) fat‐7::GFP expression in WT and *str‐2(ok3148)* animals at 15, 20, and 25°C. Scale bar, 200 µm. *p < .05, **p < .01, ***p < .001

We studied the metabolic adaptation of *C. elegans* to temperature by studying fatty acid composition, LD levels, and expression of lipid metabolism genes at high (25°C) versus low (15°C) temperature. To our surprise, we found that WT animals had less than 50% ORO staining at 15°C than at 25°C (Figure 4a,b). The decline in ORO intensity was severe and has not been reported previously for *C. elegans*. The *str‐2* animals had fewer droplets than WT animals at 25°C but they had slightly higher ORO staining than WT animals at 15°C (Figure 4b) suggesting that STR‐2 positively regulates fat storage only at higher temperature of 25°C but not at 15°C.

The fatty acid profile of WT animals at 15 and 25°C showed several changes in the fatty acid composition (Figure S6A). Abundance of branched chain fatty acids (15iso and 17iso) increased with temperature in WT animal. Among the MUFAs, palmitoleic acid 16:1 abundance declined (3.6% at 15°C vs. 2.1% at 25°C) while oleic acid 18:1n−9 abundance increased (4.4% at 15°C vs. 5.7% at 25°C) at higher temperature (Figure S6A). The magnitude of decline in polyunsaturated fatty acid (PUFA) species at 25°C was much smaller though significant for eicosatetraenoic acid (20:4n−3) and eicosapentaenoic acid (20:5). Interestingly, two PUFAs—18:2 and 20:3—showed increase in abundance at 25°C compared to 15°C (Table 1; Figure S6). The *str‐2* animals also showed changes in fatty acid composition due to temperature, similar to WT (Figure S6B). We examined the difference in fatty acid abundance in WT and *str‐2* animals, at 15 and 25°C (Table 1). Since MUFAs are known to extend *C. elegans* lifespan, we closely examined their relative levels (Han et al., 2017; Lee et al., 2019). At 25°C, the *str‐2* animals had lower levels of MUFAs—palmitoleic acid and oleic acid (18:1n−9)—than the WT (Table 1; Figure 4c,e). The *str‐2* animals had slightly lower level of saturated fatty acid 16:0 than WT animals at 15°C but slightly higher levels of 16:0 than WT at 25°C (Table 1; Figure 4d). At 25°C, the ratio of MUFA to saturated fatty acid (16:1/16:0) was significantly lower in *str‐2* animals than in WT animals and the ratio was restored in *str‐2* complementation strain (Figure 4f).

**TABLE 1:** Fatty acid analysis. Significance value () is provided for wild‐type (WT), and complemented strains against *str‐2* strain by unpaired “*t* test” at 15 and 25°C separately

Fatty acid	WT (*n* = 4)	*str−2(ok3148) *(*n* = 4)	*str−2(ok3148);* Ex[*Pstr−2::str−2*] (*n* = 5)	*str−2(ok3148);* Ex[*Pgpa−4::str−2*] (*n* = 5)
Lipid analysis at 15°C				
15iso	4.5 ± 0.3 (ns)	4.3 ± 0.2	5.3 ± 0.04 (***)	5.5 ± 0.1 (***)
16:0	8 ± 0.2 (*)	6.5 ± 0.4	7.3 ± 0.8 (ns)	5.8 ± 0.1 (ns)
17iso	4.8 ± 0.3 (ns)	4.6 ± 0.1	5.1 ± 0.1 (*)	5.1 ± 0.1 (**)
16:1	3.6 ± 0.1 (***)	2.7 ± 0.1	3.1 ± 0.1 (*)	2.9 ± 0.03 (ns)
18:0	10.3 ± 0.7 (ns)	10.3 ± 0.9	10.7 ± 0.6 (ns)	9.8 ± 0.4 (ns)
18:1n−9	4.4 ± 0.4 (ns)	4.3 ± 0.1	6.1 ± 0.1 (****)	6.1 ± 0.1 (****)
18:1n−7	26.8 ± 0.7 (ns)	25.7 ± 0.9	22.5 ± 0.2 (ns)	22.5 ± 0.2 (**)
18:2	9.2 ± 0.6 (ns)	10.5 ± 0.21	9.6 ± 0.36 (ns)	10.7 ± 0.1 (ns)
20:3	3.7 ± 0.4 (ns)	3.4 ± 0.1	3.3 ± 0.1 ns	3.8 ± 0.1 (*)
20:4	< 0.4% (*)	0.9 ± 0.3	0.5 ± 0.3 (ns)	1.04 ± 0.3 (ns)
20:4n−3	5.7 ± 0.3 (ns)	5.4 ± 0.2	4.7 ± 0.2 (ns)	5.5 ± 0.1 (ns)
20:5	16.8 ± 0.5 (*)	19.4 ± 0.6	18.3 ± 0.5 (ns)	19.9 ± 0.1 (ns)

	WT (*n* = 4)	*str−2(ok3148) *(*n* = 5)	*str−2(ok3148);* Ex*[Pstr−2::str−2]* (*n* = 4)	*str−2(ok3148);*Ex *[Pgpa−4::str−2]* (*n* = 4)
Lipid analysis at 25°C				
15iso	9.7 ± 0.2 (**)	8.7 ± 0.1	8.7 ± 0.2 (ns)	7.7 ± 0.3 (*)
16:0	8.5 ± 0.02 (**)	8.8 ± 0.1	8.9 ± 0.2 (ns)	8.3 ± 0.3 (ns)
17iso	9.4 ± 0.2 (***)	8.1 ± 0.1	7.9 ± 0.2 (ns)	7 ± 0.4 (*)
16:1	2.1 ± 0.01 (*)	1.9 ± 0.04	2.3 ± 0.1 (**)	2.3 ± 0.1 (**)
18:0	8.7 ± 0.4 (ns)	8.9 ± 0.2	9.7 ± 0.1 (ns)	9.6 ± 0.1 (ns)
18:1n−9	5.7 ± 0.1 (*)	5.2 ± 0.1	5.5 ± 0.1 (*)	4.7 ± 0.3 (ns)
18:1n−7	16.9 ± 0.2 (ns)	17.6 ± 0.2	17.8 ± 0.6 (ns)	16.6 ± 0.5 (ns)
18:2	11.8 ± 0.3 (ns)	11.4 ± 0.2	9.6 ± 0.4 (**)	11.2 ± 0.2 (ns)
20:3	3.4 ± 0.1 (*)	3.7 ± 0.1	4.1 ± 0.1 (**)	4.5 ± 0.2 (**)
20:4	2.1 ± 0.03 (*)	2.3 ± 0.1	2.5 ± 0.1 (ns)	2.7 ± 0.3 (ns)
20:4n−3	4.3 ± 0.3 (ns)	4.4 ± 0.2	5.3 ± 0.4 (ns)	4.5 ± 0.1 (ns)
20:5	15.3 ± 0.2 (**)	16.7 ± 0.2	16.3 ± 0.1 (ns)	17.7 ± 0.7 (ns)

We examined if temperature regulated expression of enzymes involved in lipid synthesis. We found that WT animals showed threefold increase in *dgat‐2*, fourfold increase in *fat‐5*, twofold increase in *fat‐6,* and sixfold decline in *fat‐7* transcript at 25°C compared to 15°C (Figure 4g–i), in agreement with previous reports for *fat‐7* transcript (Ma et al., 2015). *str‐2* animals also showed lower levels of transcripts for *fat‐5, fat‐7,* and *dgat‐2* at 20°C (Figure S4B). To confirm the temperature‐dependent effect of STR‐2 GPCR on desaturase expression, we examined expression of fat‐5::GFP and fat‐7::GFP in WT and *str‐2* animals at 15, 20, and 25°C. *str‐2* had no effect on fat‐5::GFP or fat‐7::GFP expression at 15°C (Figure 4m,n; Figure S4C,D). We observed that fat‐5::GFP expression was lower in *str‐2* compared to WT at 25°C (Figure 4m; Figure S4C). We also observed reduction in fat‐7::GFP expression in *str‐2* animals compared to WT at 20 and 25°C (Figure 4n; Figure S4D). These findings suggest that a certain threshold of MUFAs—such as palmitoleic acid and oleic acid—must be maintained at 25°C. However, *str‐2* animals were unable to upregulate *fat‐5, fat‐6, dgat‐2, or lipl‐3* at 25°C (compared to 15°C) like WT animals (Figure 4), suggesting that STR‐2 maintains MUFA levels at higher temperature.

In all, analysis of fat stores, fatty acid compositions, and gene expression analysis at different temperature across WT, *str‐2,* and complementation strains supported the requirement for STR‐2 activity at high temperature for metabolic adaptation and LD homeostasis.

### 2.5 Total stored fat contributes to longevity phenotype of *str‐2* animals

**FIGURE 5:**
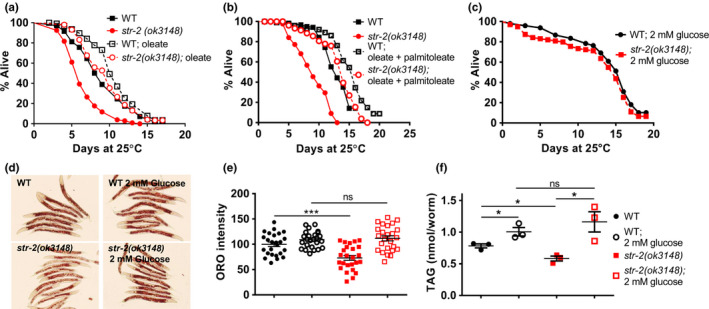
Total stored lipid content contributes to longevity of *str‐2* animals. Kaplan–Meier survival curves for wild‐type (WT) and *str‐2(ok3148)* animals (a) on *Escherichia coli* diet supplemented with 0.8% oleate, (b) on *E. coli* diet supplemented with 0.8% oleate and 0.8% palmitoleate, (c) on *E. coli* OP50 diet supplemented with 2 mM glucose. (d) Lipid stores stained by Oil Red O (scale bar 200 µM) and (e) quantification of lipid stores in WT and *str‐2(ok3148)* animals with and without glucose supplementation on OP50 diet at 25°C. Each dot represents data from individual animal. (f) Biochemical quantification of TAG in WT and *str‐2 (ok3148)* animals with and without supplementation with 2 mM glucose. *p < .05, **p < .01, ***p < .001. See Table S2 for mean lifespan and statistics

The shortened lifespan of s*tr‐2* animals on OP50 diet at 25°C could result from lower levels of total stored lipids or due to decrease in MUFA content or both. To distinguish between these two possibilities, we sought to rescue these phenotypes by dietary supplementation of MUFAs or by restoring total stored fat. Consistent with a role for MUFAs in longevity (Han et al., 2017), supplementation of 0.8% oleate increased the lifespan of *str‐2* animals and of WT animals (Figure 5a); however, it could not extend the lifespan of *str‐2* animals to that of WT animals supplemented with oleate. To test if more than one MUFA is required, we supplemented the diet with both oleate and palmitoleate. Combined MUFA supplementation greatly increased the lifespan of both WT and *str‐2* animals (Figure 5b); however supplemented *str‐2* animals were still shorter lived than supplemented WT animals. This indicated that deficiencies other than MUFAs alone contributed to shortened lifespan of *str‐2* animals. To examine the effect of total lipids, we supplemented the diet of *C. elegans* with glucose which increases LD content in *C. elegans*. We found that supplementation of diet with 2 mM glucose increased the mean lifespan of *str‐2* and WT animals to 15 days at 25°C (Figure 5c). Importantly, supplementation of 2 mM glucose increased the neutral lipid content of WT and *str‐2* animals to same level when assessed using ORO staining (Figure 5d,e) or quantified biochemically (Figure 5f). It is important to note that dietary supplementation of 2 mM glucose had a larger positive impact on longevity than combined MUFA supplementation suggesting that total fat could be an important determinant of longevity in nematodes.

Lipid droplet accumulation in *C. elegans* is linked to strain of *E. coli* used as diet (Brooks, Liang, & Watts, 2009) such that worms fed on *E. coli* HT115 have less lipids than the worms fed on *E. coli* OP50. *str‐2* RNAi using HT115 strain in *rrf‐3* animals has been reported to lead to slightly longer lifespan (Alcedo & Kenyon, 2004). Indeed, *str‐2* animals on *E. coli* HT115 diet had a small increase in lifespan compared to WT (Figure S7A). We tested if the HT115 diet could affect STR‐2‐regulated health parameters and LD homeostasis. Pharyngeal pumping rates and body length declined in WT animals on HT115 diet compared to the OP50 diet (Figure S7B–D). Interestingly, the body length and pharyngeal pumping rate differences between WT and *str‐2* animals were less pronounced on HT115 suggesting that STR‐2 does not regulate these parameters on HT115 diet. We hypothesized that the HT115 diet affects LD homeostasis and desaturase expression in such a way as to be independent of STR‐2 function. Therefore, we first asked if STR‐2‐regulated genes respond to diet change. We found that *fat‐5, fat‐6, lipl‐3, and dgat‐2* transcripts were downregulated while *fat‐7* and *acs‐2* transcripts were upregulated in WT animals on HT115 compared to OP50, suggesting that lipid synthesis was slow on HT115 (Figure S7E–J). However, *str‐2* animals had transcript levels comparable to WT on HT115. Indeed, we found that LD abundance was dramatically reduced (to <30%) in WT animals fed on HT115 than on the OP50 diet (Figure S7K,L), but WT and *str‐2* animals had similar ORO staining on HT115 diet. Altogether, the data indicate that STR‐2 does not regulate LD homeostasis and health span parameters on HT115 diet.

Taken together, analysis of lifespan and lipid homeostasis on glucose and MUFA supplementation suggests that stored fats are required for longevity in *C. elegans* in specific context of *E. coli* OP50 diet at 25°C.

### 2.6 AWC^on^ and ASI response to temperature requires STR‐2 GPCR

**FIGURE 6:**
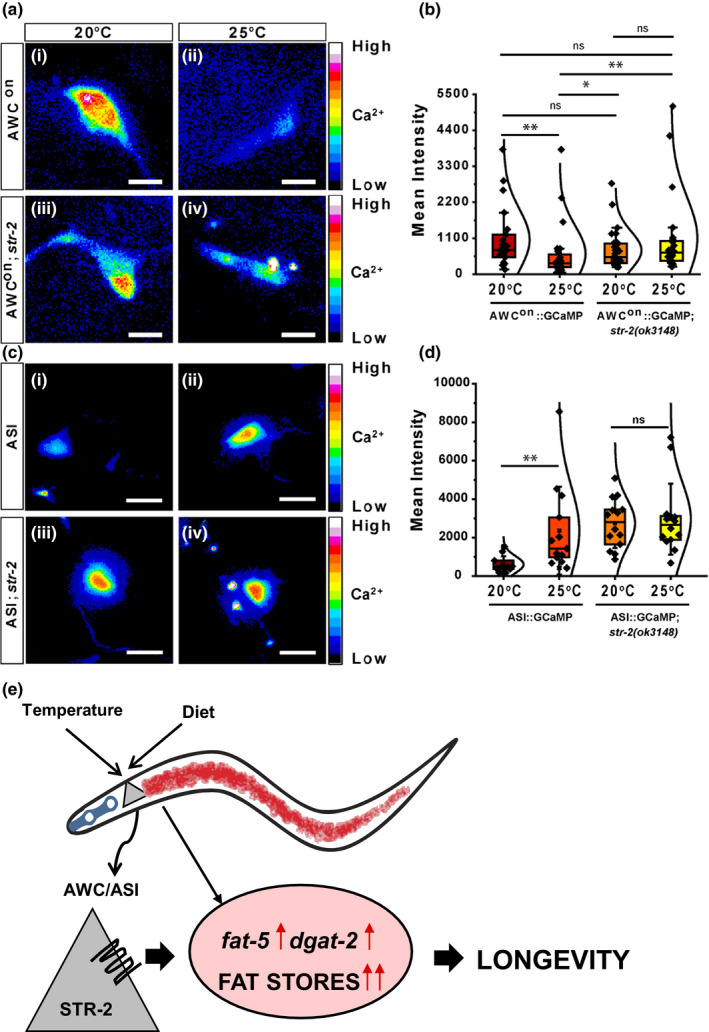
STR‐2 regulates adaptation of AWC neuron to high temperature. (a) Calcium imaging in AWC^on^ neurons. (i–iv) Pseudocolor image of GCaMP5 expressing neuron at indicated temperatures shows mean GCaMP5 intensity from average Z projections. Scale bar 5 μm. (b) Violin plot of mean GCaMP5 signal measured from the cell body in AWC^on^ in wild‐type (WT) or *str‐2(ok3148)* animals cultivated at either 20°C or 25°C. (c) Calcium imaging in ASI neurons. (i–iv) Pseudocolor image of GCaMP5 expressing neuron at indicated temperatures shows mean GCaMP5 intensity from average Z projections. Scale bar 5 μm. (d) Violin plot of mean GCaMP5 signal measured from the cell body in ASI neuron either from WT animals (i–ii) or *str‐2(ok3148)* animals (iii–iv) cultivated at 20°C (i–iii) and 25°C (ii–iv). For b and d >20, animals were imaged and presented in a violin plot with median ± inter quartile range with a normal distribution. Each dot represents data from individual animal. **p < .01, *p < .05, ns, nonsignificant. (e) STR‐2 maintains the normal lifespan of *Caenorhabditis elegans* by regulating fat storage necessary for survival at 25°C on *Escherichia coli* OP50 diet

AWC neurons exhibit stochastic calcium transients in response to change in the temperature (Kimata et al., 2012). The AWC^on^ neuron shows adaptation to increasing temperature by a decrease in cytosolic Ca^2+^ levels (Kotera et al., 2016). To test if STR‐2 regulates temperature response in the AWC^on^ neuron, we expressed GCaMP5 calcium sensor under the *str‐2* promoter and measured Ca^2+^ response. We observed a significant lowering in the basal Ca^2+^ levels in WT animals cultivated at 25°C (median 733.42; interquartile range (IQR (Q3 − Q1)) = 694.62) compared to the WT animals grown at 20°C (median 327.5; IQR (Q3 − Q1) = 389.36) (Figure 6a (i, ii), b). In contrast, *str‐2* animals grown at 20°C showed a marginal decrease in basal calcium levels than WT animals at 20°C (median 519.89; IQR (Q3 − Q1) = 597.52) (Figure 6a,b). Unlike WT animals, *str‐2* animals exposed to 25°C (median 658.76; IQR (Q3 − Q1) = 606.74) did not show any significant decrease in Ca^2+^ levels compared to *str‐2* animals grown at 20°C (Figure 6a,b). We found that the basal Ca^2+^ level in *str‐2* animals at either 20°C or 25°C was significantly higher than in WT animals grown at 25°C. This suggests that STR‐2 receptor function is required for decline in Ca^2+^ level in AWC^on^ neuron at higher temperatures necessary for adaptation.

To test if STR‐2 regulates temperature response in the ASI neuron GCaMP signal from ASI neurons were measured. At 20°C, GCaMP signal is ASI neuron that remains relatively lower (median 478.32; IQR (Q3 − Q1) = 428.881) in comparison with AWC^on^ neurons. However, at higher temperature, we observed significant increase in basal Ca^2+ ^levels in ASI neuron in WT animals (median 1,431.37; IQR (Q3 − Q1) = 2,082.40) Figure 6c (i, ii) consistent with previous observation (Beverly, Anbil, & Sengupta, 2011). Interestingly, *str‐2 *mutant animals grown at 20°C showed an increase in basal calcium levels than WT animals at 20°C (median 2,810.02; IQR (Q3 − Q1) = 1,812.134) (Figure 6c,d) which remained unchanged in *str‐2* animals grown at 25°C (median 2,668.36; IQR (Q3 − Q1) = 1,224.98) (Figure 6c (iii), (iv)). Similar to AWC^on^ neurons in *str‐2* mutant animals, no change in Ca^2+^ signal in ASI neuron in *str‐2* animals at 25°C suggests that regulated Ca^2+^ entry through STR‐2 receptor activity at higher temperature may modulate downstream signaling events, consistent with the requirement of STR‐2 function in longevity and lipid metabolism at high temperature.

Based on the findings of this study, we propose a model in which STR‐2 GPCR, via its activity in AWC and ASI neurons, plays a critical role in the regulation of lifespan of *C. elegans* at 25°C on *E. coli* OP50 diet by maintaining neutral lipid levels (Figure 6e).

## 3 DISCUSSION

Longevity of an organism is regulated by environmental factors such as temperature and diet, as well as genetic factors. *C. elegans* exhibits longer lifespan at lower temperature, while high temperature shortens its lifespan. This shortening of lifespan at high temperature has been shown to be regulated by nervous system in *C. elegans*; however, mechanism of adaption to temperature in this context is not clear. Here, we show that STR‐2, a neuronal GPCR, controls metabolic adaptation to higher temperature and thus controls lifespan of *C. elegans* at 25°C.

Response to temperature includes metabolic adaptation such that animals living in cooler areas have higher levels of PUFAs while those living in hot regions have high saturated fat: unsaturated fat ratio. Fatty acids composition regulates membrane fluidity and may also regulate cellular signaling. Metabolic alterations play a key role in the modulation of aging in mammals as well as *C. elegans* (Solon‐Biet et al., 2014; Xiao et al., 2015). A recent report also shows MDT‐15 dependent changes in unsaturated fatty acids, MUFAs and PUFAs (Lee et al., 2019) at 15 and 25°C. The fatty acid profile between their study and ours is similar but with small differences in levels of palmitoleic acid and oleic acid. They also report increased *fat‐5* transcript expression at high temperature but no change in *fat‐6* transcript levels at increased temperature. Long‐lived mutants such as *daf‐2* and *glp‐1* show an increase in fat stores which suggests that total fats or certain kinds of stored fat might be beneficial for worms, possibly to counter stress (Amrit et al., 2016; O'Rourke, Soukas, Carr, & Ruvkun, 2009). On the other hand, mutations in stearic acid desaturases (*fat‐6, fat‐7*) lead to reduced LDs and lower brood size (Brock et al., 2007). Age‐dependent decline in desaturase expression has also been reported in mice (Kumar et al., 1999). Additionally, desaturases are upregulated in long‐lived *daf‐2* and histone methyltransferase‐deficient worms (Halaschek‐Wiener et al., 2005; Han et al., 2017). MUFAs are key components of phospholipids, triglycerides, and cholesterol esters. They are beneficial for humans, and dietary supplementation of MUFA increases lifespan in *C. elegans*. Consistent with these observations, we found that *str‐2* mutant animals with fewer LDs than WT were susceptible to heat stress and were short lived. Additional examples of GPCR‐regulated LD homeostasis have also been reported in large scale screen or serotonin signaling studies (Ashrafi et al., 2003) suggesting that many GPCRs can regulate lipid homeostasis. BAG neurons, via an unknown sensory mechanism or GPCR, can regulate satiety response and LD abundance in *C. elegans* (Juozaityte et al., 2017) providing further support for neuronal regulation of LD homeostasis.

Why is STR‐2 activity dispensable for *C. elegans* at lower temperature of cultivation or on HT115 diet? Common features between these two conditions include lower pharyngeal pumping rates and fewer LDs in the intestine. On OP50 diet at 25°C, STR‐2 regulates pharyngeal pumping. Analysis of the connectome in *C. elegans* hermaphrodite indicates that AWC neurons, expressing STR‐2, are capable of stimulating contraction of pharyngeal muscles to regulate pumping although none of the circuits have been studied. Lower pumping rates at 15°C can cause slower buildup of calorie excess in the intestine and, therefore, slower rate of LD synthesis. Lipid utilization and fatty acid beta oxidation may also be slow at low temperature. Another interesting question is why fat storage in the intestine requires neuronal regulation on OP50 diet at 25°C. The answer may lie in the increased abundance of LDs in this scenario. Altered diet and higher temperature can enhance pharyngeal pumping, can induce desaturase and DGAT‐2 expression leading to more LDs as shown in this study. The expression of lipid synthesis enzyme above a threshold appears to require noncell autonomous inputs from the nervous system (STR‐2) for a speedy response.

Serotonin signaling is known to regulate lipid metabolism, although the identity of specific neurons and specific stimulus (other than feeding itself) remains unknown (Murakami & Murakami, 2007; Srinivasan et al., 2008). Recently, glutamate and serotonin have been implicated in the IL1 sensory neuron cooling circuit and insulin‐like peptides have been implicated in the ASJ‐dependent warming circuit for longevity regulation (Zhang et al., 2018). Many mutations affecting AFD thermosensory neuron or the circuit, *ttx‐1, gcy‐8, daf‐9*, regulate longevity at 25°C as shown earlier (Lee & Kenyon, 2009) but did not affect LD homeostasis in our analysis (data not shown). It would be interesting to examine if regulators of metabolic adaptation to temperature such as ACDH‐11 regulate lifespan. STR‐2, being a GPCR with known neuronal expression, provides an opportunity to study AWC and ASI neural circuits for metabolic adaptation. The identification of the neurotransmitter or neuropeptide utilized by the STR‐2‐dependent longevity circuit would be an interesting follow‐up of the current study. Lipid metabolism is regulated by several transcription factors including SREBP/SBP‐1, HLH‐30, NHR‐49, and NHR‐80 (Brock, Browse, & Watts, 2006; Van Gilst, Hadjivassiliou, Jolly, & Yamamoto, 2005; Watts, 2009), by transcriptional coactivator MDT‐15 (Taubert, Van Gilst, Hansen, & Yamamoto, 2006), and by chromatin modifiers (Han et al., 2017). STR‐2 might regulate one of these, or possibly a novel, context specific factor in the intestine.

Dramatic changes in LD homeostasis in *C. elegans* due to temperature and diet change, shown in this study and earlier (Brooks et al., 2009; Xiao et al., 2015), provide exciting avenues for research on longevity and metabolism in *C. elegans*, fruit fly, and mammals. In social insects, such as ants and bees with caste systems, queens and workers have strikingly different diets and the queen lives far longer than the workers (Page & Peng, 2001). Could we discover neuronal circuits in other organism to bring about changes in MUFA levels, stored fats, and longevity? OP50 versus HT115 diet affects longevity phenotypes differently for *str‐2* (this study), *rict‐1*, *alh‐6* (Pang & Curran, 2014; Soukas, Kane, Carr, Melo, & Ruvkun, 2009), suggesting that lifespan studies on different diets should be equated with caution. At the same time, these differences provide us with opportunity to find diet‐specific genetic and pharmacological regulators of lipid metabolism in *C. elegans* and other animals.

## 4 EXPERIMENTAL PROCEDURES

### 4.1 *C. elegans* and *E. coli* strains

*C. elegans* were cultured using standard techniques (Brenner, 1974). The Bristol WT strain was used as WT. WT, VC2413 [*str‐2(ok3089)*], RB2316 [*str‐2(ok3148)*], AH159 [*sra‐13(zh13)*], and CX32 [*odr‐10(ky32)*] strains were obtained from the Caenorhabditis Genetics Center (CGC) University of Minnesota, Minneapolis. VSL1502 [agEx(*str‐2p*::GCaMP5A + *unc‐122p*::GFP) and *str‐2(ok3148);* agEx(*str‐2p*::GCaMP5A + *unc‐122p*::GFP) (tt1701) were created by crossing. Transgenic strains VSL1701 [adEx(*str‐2(ok3148);Pstr‐2::str‐2;unc122p*::dsRed)], VSL1702 [adEx(*str‐2(ok3148);Pgpa‐4::str‐2;unc122p*::dsRed] and VSL1703 [adEx(WT*;Pstr‐2::str‐2;unc122p*::dsRed)] and VSL1706 (*str‐2(ok3148);fat‐7::gfp)* double mutants were created in the laboratory. The strains *str‐2(ok3089)* and *str‐2(ok3148)* were outcrossed three times to WT to remove any background mutation and renamed as VSL1510 and VSL1511. *E. coli* strains OP50 and HT115 (DE3) obtained from CGC.

### 4.2 Culture conditions

*E. coli* strains were grown overnight in Luria Broth at 37°C. Nematode growth medium (NGM) agar plates containing 50 µg/ml streptomycin (for *E. coli* OP50) or 12.5 µg/ml tetracycline (for *E. coli* HT115) were seeded with 0.2 ml of overnight grown bacterial culture.

### 4.3 Construction of transgenic and double mutant animals

Transgenic animals were constructed by standard cloning and microinjection techniques. 1.6 kb full‐length *str‐2* genomic DNA was inserted into vector pPD95.77 SL2 GFP. 2 kb *str‐2* promoter or 2.7 kb *gpa‐4* promoter was cloned. Transgenic lines were obtained by injecting plasmid (50 ng/μl) with the *unc‐122p::ds red* marker plasmid (50 ng/μl). VSL2001(*str‐2*(*ok3148*);FAT‐5::GFP) strain was generated by crossing *str‐2(ok3148)* males with *waEx15* hermaphrodites as per the standard protocol (Fay, 2006). VSL2001(*str‐2*(*ok3148*);fat‐5::GFP) strain was generated by crossing *str‐2(ok3148)* males with BX150 [waEx18(fat‐5::GFP + lin‐15(+)] hermaphrodites as per the standard protocol (Fay, 2006). VSL2002(*str‐2*(*ok3148*);fat‐7::GFP) strain was generated by crossing *str‐2(ok3148)* males with DMS303 [nIs590(fat‐7p::fat‐7::GFP + lin‐15(+)] hermaphrodites. Primer sequences for cloning and genotyping are mentioned in Table S1.

### 4.4 Lifespan assay

The lifespan of worms of indicated genotype was assayed at 15, 20, and 25°C on 60 mm NGM plates seeded with *E. coli* culture (Lee, Tian, Grosmaitre, & Ma, 2009). Worms were synchronized to YA stage at indicated temperature or diet and transferred to plates containing 5‐fluoro‐2′‐deoxyuridine (FUdR) (20 µM; Sigma‐Aldrich) unless stated otherwise. All the lifespan assays in Figure 1 were done in NGM plates without FUdR. The number of dead worms was counted each day, and live worms were transferred to fresh NGM plates every third day. Animals that crawled off the plate or exploded were excluded from analysis. 0.8% sodium oleate (Sigma‐Aldrich) or 0.8% each of sodium oleate and sodium palmitoleate in 0.001% NP40 was supplemented as described earlier (Han et al., 2017). For glucose supplementation, eggs were allowed to develop on OP50 plates with and without 2 mM glucose at 25°C and treatment was continued through the lifespan assay. p values were obtained using the log‐rank (Mantel–Cox) method. Mean TD^50^ values and number of animals used for each assay are provided in Table S2. We found that the lifespan of WT animals in our laboratory was on the lower side of reported values (Table S2). Therefore, we performed lifespan analysis at the entire range of rearing temperature (15, 20, and 25°C). We found that there was negative correlation between temperature and lifespan on WT animals (Figure S8) as reported by many laboratories earlier (Lee & Kenyon, 2009).

### 4.5 Oil Red O staining

LDs were stained using Oil Red O staining method as described earlier (O'Rourke et al., 2009). Synchronized worms grown at indicated temperature (or diet) were stained with ORO before imaging at 10× or 20× magnification using an Olympus IX81 inverted microscope. ORO intensity was analyzed through ImageJ. (NIH). 10–30 worms under each condition were used for quantification.

### 4.6 Fatty acid composition analysis of *C. elegans*

To measure fatty acid composition, approximately 400 YA stage worms (containing 0–8 embryos) were washed from feeding plates with water on ice and washed once to remove residual bacteria. After settling on ice again, as much water as possible was removed (~90%). Fatty acids were converted to methyl esters by incubating worm suspensions for 1 hr at 70°C in 2 ml of 2.5% sulfuric acid in methanol. Following incubation, the reactions were stopped by adding 1 ml of water and then mixed thoroughly with 200 μl of hexane to extract the resulting fatty acid methyl esters. We measured relative amount of fatty acid methyl esters by injecting 2 μl of the hexane layer onto an Agilent 7,890 GC/5975C MS in scanning ion mode equipped with a 20 × 0.25 mm SP‐2380 column (Supelco).

### 4.7 Pharyngeal pumping

10–20 worms developed at indicated temperature and diet were used for this assay. Pharyngeal contractions of YA worms in 1 min were assayed under stereoscope.

### 4.8 Brood size and body length

Brood size was measured at 25°C as reported (Shomer et al., 2019; Soukas et al., 2009). Single late L4 stage worms (10 worms per strain) were kept on individual OP50‐seeded plates. The animals were transferred to new plate every 24 hr until they stopped producing progeny. Total brood size was determined as the sum of progeny produced by a single worm.

For body length measurement, synchronized YA worms of each strain at indicated temperature and diet were imaged and length was measured using ImageJ software (NIH). 15–20 worms for each condition were used for the length measurement.

### 4.9 RNA isolation and qPCR

Worms were synchronized and allowed to develop at indicated temperature and diet. Worms were harvested in TRIzol and RNA was isolated using RNeasy Plus Universal Kit (Qiagen). cDNAs were synthesized using iScript Reverse Transcription System (Bio‐Rad) and subjected to real‐time PCR analysis using the SYBR Green Supermix (Bio‐Rad) on QuantStudio 3 Real‐Time PCR system (Thermo Scientific). Fold change of dysregulated genes is obtained by formula 2^−(ΔΔCt)^ and of upregulated genes by formula −1/2^−(ΔΔCt)^ (Schmittgen & Livak, 2008). Primer sets used are shown in Table 1. *act‐1* was used as control gene for normalization. 2–6 biological replicates were used for qPCR analysis.

### 4.10 Heat stress and oxidative stress assays

YA worms of WT and mutant strain synchronized at 20°C were used for heat stress assay at 34°C, and the number of dead worms was counted each hour. For oxidative stress assay, worms were transferred to NGM plates containing 20 mM paraquat and the number of dead worms was counted every 4 hr (Park, Tedesco, & Johnson, 2009). Animals that crawled off the plate or exploded were excluded from analysis. Graphs were plotted, and TD50 was calculated as mentioned for lifespan assay. Mean TD50 values and number of animals used for each assay are given in Table S2.

### 4.11 Microscopy

The 3‐ and 6‐day‐old adult worms were visualized for lipofuscin accumulation under DAPI filter with excitation at 358 nm and emission at 461 nm. Relative fluorescence intensity was determined by measuring average pixel intensity using ImageJ software (NIH).

Imaging for fat‐5::GFP and fat‐7::GFP strains was done using LEICA DMi8 microscope at room temperature. Quantification of GFP expression was done in 18 to 32 animals for every strain/condition using ImageJ.

### 4.12 Total Lipid quantification

Triacylglycerol quantification was done by a fluorometric assay using BioVision kit (catalog # K622‐100). Briefly, YA worms (1,000–2,000) were harvested in water containing 5% of NP‐40, washed 3 times, and sonicated in 500 µl 5% NP‐40 in water at maximum amplitude, for 30 min on ice. Samples were heated to 80–100°C for 2–3 min and cooled down at room temperature, twice. Samples were centrifuged at 15,000 g for 2 min to remove insoluble material. Supernatant was diluted up to 1:10,000 before measurement. A standard curve was generated using known concentration of TAG. TAG per well for each *C. elegans* sample was calculated from the standard curve. Total TAG was quantified by multiplying TAG per well with dilution factor. Total TAG in the initial volume (500 µl) was divided by the total number of worms per sample to obtain TAG/worm.

### 4.13 Calcium Imaging and Image processing

Calcium imaging was performed in Olympus IX83 microscope with Perkin Elmer Ultraview Spinning Disk unit and a Hamamatsu EMCCD camera. Animals in L2 and L3 larval stage were picked and cultivated at 20°C or 25°C for >12 hr till they become 1 day old. Just before imaging worms were anesthetized in 5 mM Tetramisole (preheated to 25°C for imaging at higher temperature) (Sigma‐Aldrich) and mounted on 5% agarose pad that was kept inside a closed heated chamber maintained at either 20°C or 25°C (Tokai Hit). The temperature during the imaging session was constantly monitored by a thermocouple temperature sensor. GCaMP5 was excited using a 488 nm laser (10% laser intensity was fixed for all the conditions) with 400 ms exposure, EM gain 5, and detector sensitivity was kept at 190. 3D stacks were acquired with 300 nm spacing and merged using average Z projection function embedded in ImageJ. Mean intensity of the cell body was measured after a background subtraction from an area closer to the cell body and represented as a scatter plot. Median and IQR were used to report the change in calcium levels.

### 4.14 Statistics

Lifespan and stress assays were analyzed using the log‐rank (Mantel–Cox) test. *p* < .05 was considered significant (see Table S2). Pharyngeal pumping, body length, ORO intensity, GFP intensity, and fold change (qPCR) were presented as mean ± *SEM* and analyzed using unpaired Student's *t* test. For calcium imaging experiments, nonparametric test between two independent samples was performed and significance was calculated by both Mann–Whitney test.

## CONFLICT OF INTEREST

The authors declare no conflict of interest.

## AUTHOR CONTRIBUTIONS

AD, AS, SM, MS, and JLW performed the experiments. AD, AS, SM, MS, JLW, and VS analyzed the data. AD, AS, SM, SPK, JLW, and VS conceptualized the study and wrote the manuscript.

## Supporting information

Supplementary Material

## Data Availability

The raw data are available in a dataset at Mendeley (Singh, 2019). It is also accessible at https://data.mendeley.com/datasets/85h37zd9r6/1.
