# Supplementary material for: Neuronal control of lipid metabolism by STR‐2 G protein‐coupled receptor promotes longevity in Caenorhabditis elegans
Source: Aging Cell. 2020 May 20;19(6):e13160. doi: 10.1111/acel.13160 (PMC7294788; doi:10.1111/acel.13160)
Supplement: Supplementary file 1 — Supplementary Material [file ACEL-19-e13160-s001.docx]

Table S1: Sequences of primers used in the manuscript

| S.No. | gene | Primer sequence |
| --- | --- | --- |
|  | qPCR | |
| 1 | *fat-1* | GAAGGCTAAGGAGTTCGTGC  CCATCCTTATATTTCAGCCTC |
| 2 | *fat-2* | ATATTGAGGTCTACGAAGCTG  GTCAAATAGTCGAGGGTAACG |
| 3 | *fat-3* | TTTGATTGCTCACGTAGTCAC  CGTCAAAGTAGTCATCGACG |
| 4 | *fat-4* | TGTTTCTCATCTTGTTGGAGG  GGTAAACCATTTGCTGCTGC |
| 5 | *fat-5* | TCTACACATTGGAGCCCTG  CAGGAGGAATACCCATCCA |
| 6 | *fat-6* | CAACTTCCATCACACATTCCC  TCCTCGTTGAATATCACATCC |
| 7 | *fat-7* | GGAAGGAGACAGCATTCATTGCG  GTCTTGTGGGAATGTGTGGTGG |
| 8 | *elo-1* | GTTACTATTCAGGCTTCAATCCTG  TTGGAATGGTTGACGATTACG |
| 9 | *elo-2* | TCACTGTTCTCGGGAATCG  AGTAGGAAGCGACAAACCC |
| 10 | *fasn-1* | GGATAATACTGGAGAAGGATCG  ATGTTGTCCGAAGACTGAG |
| 11 | *pod-2* | GAGCAGTATTACGAGACGC  GAGCTGCTGTTCATATCGT |
| 12 | *lipl-1* | GGACTTAAAGTTGAAGCTGGAG  AACACGAGTTGCGTTAAGC |
| 13 | *lipl-2* | GTTACATGGCCAAATGGGA  AAACGAAAGCTGCACTCTG |
| 14 | *lipl-3* | ATGGGCAGGCAAATCCACCA  AGTTGTTCTGCGCAATTATA |
| 15 | *lipl-4* | ATGGCCGAGAAGTTCCTACATCGT  GGTGAATTGGCGACCCAATCGAAA |
| 16 | *lipl-5* | CAAGAAGTTCTTCGCGCTG  CGAACCATCCATCAAACTCC |
| 17 | *lipl-7* | GACAGCGAGTGTGGATTTGG  GTATGGAAGTCGACCGAGCAAG |
| 18 | *hosl* | CAAAGTTTGGCGGACACAGC  CAGGGAGGCAACTGGCAC |
| 19 | *pla2* | CTGTGATACACTAAGTGAGGC  CTGCAATTTCAGCAAAAACCG |
| 20 | *atgl-1* | GATCGACCGATGATTTATCGAG  GAGCCAATCCACATTTGGT |
| 21 | *dgat-2* | TCGGATATCATCCTCATGGA  ATGCCTTTGAACACTTGCT |
| 22 | *ech1.1* | GATATAGCCTCTGTATTTGGTC  AATTGTCCTCCACTCTTCGC |
| 23 | *acs-1* | AAGGAAAAACGACTGAAGAGG  GACGACTTGCTGGAGACCC |
| 24 | *acs-2* | TCCGGATAAGGAGTTCTGTG  ATTTGACGGACGTCATGGT |
| 25 | *acs-4* | AAACAACTCGAAGAACATGCC  TGCTTATACAGATCATCGAGG |
| 26 | *acs-5* | ATTCGAAGCTCCTGGATTGG  CATTATTCTCCATTCTCATCAC |
| 27 | *acs-13* | CGTCAAAAAGGCGATCCTCG  GTTGCTTCGAGTAGTGAGCC |
| 28 | *acs-15* | GAGGGAAAGACCTACGAGG  GAACTGTGCAATTGCAGTCC |
| 29 | *acs-16* | CACGGCAGTTTGTGTCGCC  GCTCTCCAGCATGGCGCG |
| 30 | *acs-17* | GCATTGCAAAAGGAGTTGGC  CAAGTTCTTTGCGGAAGGCC |
| 31 | *acs-18* | GCAGATGATGTGCTGCGAC  CTTATACATTCCATCAATTTCCG |
| 32 | *acs-22* | TTGAATCAGGTGAAACAGCA  CAATTCTTGATACGTCAACTGC |
|  | Gene complementation | |
| 33 | *str-2p Pst1* | AACTGCAGCTTCGCTGCCCCAATT  CCCTGCAGCAGTCGGCATTTTTAT |
| 34 | *str-2 gene Pst1* | AACTGCAGATGCCGACTGTGCAAT  ACTCCCGGGTTAATTTGTCACGTA |
| 35 | *gpa-4p Pst1* | AACTGCAGGATCATTGGAAATGCG  TCCTGCAGTGTTGAAAAGTGTTC |
|  | Genotyping | |
| 36 | *str-2 (ok3148)* | GACGTTGCAAAGATTTAATG  TATTATGGAGAAACGCCG |
| 37 | *str-2 (ok3089)* | CATAGCAGCAGGCATGTAC  GCTCAGCAACTACCGATTG |

**Table S2:** Life span data. Each set of lifespan analysis is associated with its corresponding figure in the text as indicated on the left of the table. Means, standard error of the mean (SEM) and P values were calculated using the log-rank test (Mantel-Cox) from Kaplan-Meier survival analysis of cumulative data obtained from the indicated numbers of animals.

| **Condition** | **Genotype** | **Mean life span**  **(days)** | **Number of animals (assayed/**  **total)** | **p value**  **(in comparison to WT)** | **Figure** |
| --- | --- | --- | --- | --- | --- |
| **No FUDR#** | | | | | |
| *E. coli* OP50, 20°C | WT  *str-2(ok3148)* | 10  6.93 | 105/110  111/115 | <0.0001 | 1(B) |
|  | WT  *str-2(ok3148)* | 10.7  7.67 | 61/97  92/103 | 0.001 |  |
|  | WT  *str-2(ok3089)* | 11.4  9.08 | 114/140  111/140 | <0.0001 | 1(A) |
|  | WT  *str-2(ok3089)* | 11  7.6 | 93/120  75/105 | <0.0001 |  |
| *E. coli* OP50, 25°C | WT  *str-2(ok3148)* | 7.62  4.77 | 133/135  141/150 | <0.0001 | 1(C) |
|  | WT  *str-2(ok3148)* | 8.5  4 | 114/115  117/120 | <0.0001 |  |
|  | WT  *str-2(ok3148)* | 9.3  6.13 | 111/120  104/110 | <0.0001 |  |
| *E. coli* OP50, 15°C | WT  *str-2(ok3148)* | 21.9  20.1 | 74/115  74/110 | ns | 1(D) |
|  | WT  *str-2(ok3148)* | 18.2  16.5 | 72/120  63/110 | ns |  |
| *E. coli* OP50, 20°C | WT  *sra-13(zh3)* | 13  13.2 | 78/120  87/120 | ns | S1(A) |
|  | WT  *sra-13(zh3)* | 11.8  12.2 | 47/70  29/65 | ns |  |
|  | WT  *odr-10(ky32)* | 10.8  10.4 | 93/120  96/125 | ns | S1(B) |
|  | WT  *odr-10(ky32)* | 11.8  11.3 | 47/70  68/130 | ns |  |
| **E. coli* OP50, 34°C | WT  *str-2(ok3148)* | 16.1 hrs  14.7 hrs | 35/45  48/50 | 0.002 | 1(F) |
|  | WT  *str-2(ok3148)* | 17.3 hrs  15.3 hrs | 34/50  40/50 | 0.0002 |  |
| **E. coli* OP50, 20°C,  Paraquat | WT  *str-2(ok3148)* | 44 hrs  44 hrs | 115/130  89/100 | ns | S2(D) |
|  | WT  *str-2(ok3148)* | 43 hrs  40.3 hrs | 56/80  68/80 | ns |  |
| *E. coli* OP50, 25°C | WT  *str-2(ok3148)*  *str-2(ok3148);Ex[Pceh-36::str-2]* | 11.3  7.27  8.87 | 127/130  138/145  96/97 | <0.0001  <0.0001 | S3(B) |
| **With FUdR (20 µM)** | | | | | |
| *E. coli* OP50, larval development at 25°C and life span assay at 15°C | WT  *str-2(ok3148)* | 30  20 | 83/100  62/100 | <0.0001 | S1(C) |
| *E. coli* OP50, 25°C | WT  *str-2(ok3148)*  *str-2(ok3148);Ex[Pstr-2::str-2]*  *str-2(ok3148);Ex[Pgpa-4::str-2]*  *str-2OE* | 9.87  6.2  10.3  9.8  9.13 | 95/98  106/108  99/105  103/110  95/103 | <0.0001  ns  ns  ns | 3(A) and S3(A) |
| *E. coli* OP50, 25°C | WT  *str-2(ok3148)*  *str-2(ok3148);Ex[Pstr-2::str-2]*  *str-2(ok3148);Ex[Pgpa-4::str-2]* | 13.8  8.87  13.6  14.5 | 122/150  136/136  136/145  156/163 | <0.0001  ns  ns |  |
| *E. coli* HT115, 25°C | WT  *str-2(ok3148)* | 12.2  13.3 | 124/130  133/135 | <0.0001 | S7(A) |
|  | WT  *str-2(ok3148)* | 10.6  14.2 | 124/130  54/58 | <0.0001 |  |
|  | WT  *str-2(ok3148)* | 12.2  12.5 | 92/100  118/127 | ns |  |
| *E. coli* OP50, 25°C, Sodium oleate | WT no supplementation  *str-2(ok3148)* no supplementation  WT oleate  *str-2(ok3148)* oleate | 8.6  6.5  9.93  9.13 | 121/123  118/123  118/130  151/161 | <0.0001  <0.0001  ns | 5(A) |
| *E. coli* OP50, 25°C, Sodium oleate and sodium palmitoleate | WT no supplementation  WT oleate and palmitoleate supplementation  *str-2(ok3148)* oleate and palmitoleate | 7.47  12.4  10.2 | 93/100  85/100  86/100 | <0.0001  <0.0001^a^ | 5(B) |
|  | WT no supplementation  WT oleate and palmitoleate supplementation  *str-2(ok3148)* oleate and palmitoleate | 6.50  10.5  10.5 | 99/100  89/100  91/100 | <0.0001  ns^a^ |  |
|  | WT no supplementation  WT oleate and palmitoleate supplementation  *str-2(ok3148)* oleate and palmitoleate | 6.5  14.6  12.3 | 99/100  92/100  98/100 | <0.0001  <0.0001^a^ |  |
| *E. coli* OP50, 25°C, 2 mM glucose  supplementation | WT no supplementation  WT 2 mM Glucose  *str-2(ok3148)* no supplementation  *str-2(ok3148)* 2 mM Glucose | 11.5  15  3  14.5 | 87/100  85/100  94/100  86/100 | <0.0001  <0.0001  ns^b^ |  |
|  | WT no supplementation  WT 2 mM Glucose  *str-2(ok3148)* no supplementation  *str-2(ok3148)* 2 mM Glucose | 11.3  13.3  6.8  12.4 | 86/100  92/100  84/100  85/100 | <0.0001  <0.0001  <0.001^b^ |  |
|  | WT no supplementation  WT 2 mM Glucose  *str-2(ok3148)* no supplementation  *str-2(ok3148)* 2 mM Glucose | 9.6  13.1  6.7  13.3 | 94/100  40/50  6.7  13.3 | <0.0001  <0.0001  ns^b^ | 5C |
| *E. coli* OP50, 15°C, 20°C and 25°C | WT at 15°C  WT at 20°C  WT at 25°C | 18.2  11  8.5 | 72/120  93/120  114/115 | <0.0001  <0.0001 |  |
|  | WT at 15°C  *str-2(ok3148)* at 15°C  WT at 20°C  *str-2(ok3148)* at 20°C  WT at 25°C  *str-2(ok3148)* at 25°C | 30  30  21.4  12.1  14.5  10.7 | 126/130  113/120  117/120  115/120  116/120  115/120 | ns  <0.0001  <0.0001 | S8 (WT only) |
|  | WT at 15°C  *str-2(ok3148)* at 15°C  WT at 20°C  *str-2(ok3148)* at 20°C  WT at 25°C  *str-2(ok3148)* at 25°C | 23  23.4  21  10.6  14.8  10 | 119/120  116/120  120/123  59/60  115/118  116/120 | ns  <0.0001  <0.0001 |  |
|  | WT at 15°C  *str-2(ok3148)* at 15°C  WT at 20°C  *str-2(ok3148)* at 20°C  WT at 25°C  *str-2(ok3148)* at 25°C | 32  32  21.1  10.7  15  8.4 | 111/120  59/60  117/120  115/120  119/120  118/120 | ns  <0.0001  <0.0001 |  |

# All survival or life span assays without FUDR are shaded in grey

* Mean survival is expressed in hours

^a^ *p-value* compared to WT Sodium oleate and sodium palmitoleate supplementation

^b^ *p-value* compared to WT 2 mM Glucose

**Supplementary Figure Legends**

**Figure S1:** **ODR-10 and SRA-13 GPCR do not regulate life span.** Kaplan-Meier survival curves of wild type N2 (WT) animals along with (A) *sra-13(zh3)*, and *(B) odr-10(ky32),* at 20°C. (C) Kaplan-Meier survival curves of WT and *str-2(ok3148)* animals with larval development at 25°C and life span at 15°C. See also Table S2 for lifespan conditions and statistics.

**Figure S2:** **Effect of *str-2* mutation and temperature on health span parameters.** (A and B) Lipofuscin accumulation in WT and *str-2* animals measured by epifluorescence in DAPI channel. (C) Total brood size of WT and *str-2* animals. Kaplan Meier survival curve of (D) WT and *str-2* animals on 20 mM paraquat (ns). (E) WT and *str-2* animals on *P. aeruginosa* PA14 (ns) (F) WT and *str-2* animals on *Enterococcus faecalis* OG1RF (ns). Log Rank test was used for D-F. (G) Pharyngeal pumping of worms grown at 15°C or grown at 25°C. (H) Comparison of body length of worms grown at 15°C or grown at 25°C. *=p < 0.05, **=p < 0.01, *** = p < 0.001, ns=nonsignificant.

**Figure S3: Overexpression of *str-2* does not increase *C. elegans* life span.** Kaplan-Meier survival curves for (A) wild type WT, *str-2(ok3148)* and WT; Ex[P*str-2*::*str-2*] animals and (B) wild type WT, *str-2(ok3148)* and WT; Ex[P*ceh-36* ::*str-2*] animals at 25°C. See also Table S2 for lifespan conditions and statistics.

**Figure S4:** **Regulation of lipid metabolism by GPCR STR-2.** (A) qPCR analysis of lipid metabolism genes in wild type WT and *str-2(ok3148)* animals at 25°C fed with *E. coli* OP50. (B) qPCR analysis of *fat-5, fat-6, fat-7, dgat-2, lipl-3 and acs-2* genes in wild type WT and *str-2(ok3148)* animals at 20°C fed with *E. coli* OP50. (C) Quantification of GFP expression in fat-5::GFP and fat-5::GFP;*str-2(ok3148)* at 15°C, 20°C and 25°C. (D) Quantification of GFP expression in fat-7::GFP and fat-7::GFP;*str-2(ok3148)* at 15°C, 20°C and 25°C. *=p < 0.05, **=p < 0.01, *** = p < 0.001, ns=nonsignificant.

**Figure S5:** **Lipid metabolism changes in aged animals.** (A) Visualization of lipid droplets in 3d old WT and *str-2(ok3148)* adults. (B) Quantification of lipid droplet stores in young adult and 3d old adults of WT and *str-2*. (C) qPCR analysis of *fat-5, fat-6, fat-7, dgat-2, lipl-3 and acs-2 in* young adult and 3d old WT adults and in young adult and 3d old *str-2* adults. *=p < 0.05, **=p < 0.01, *** = p < 0.001, ns=nonsignificant.

**Figure S6:** **Temperature regulates fatty acid composition.** Comparative analysis of individual fatty acids at 25°C and 15°C in (A) WT wild type and *str-2 (ok3148)* animals. Unpaired t test used for analysis *=p < 0.05, **=p < 0.01, *** = p < 0.001, ns= not significant.

**Figure S7:STR-2 does not regulate health span on *E. coli* HT115 diet.** (A) Kaplan-Meier survival curves for WT, *str-2* animals fed with *E. coli* HT115. (B) Pharyngeal pumping in wild type and *str-2(ok3148)* animals fed with *E. coli* OP50 or HT115. (C) Bright field images and (D) Body length measurement of WT and *str-2(ok3148)* animals. (E) qPCR analysis of STR-2 regulated lipid metabolism genes in *str-2* animals over WT animals fed *E. coli* OP50 or *E. coli* HT115. (F) qPCR analysis of STR-2 regulated lipid metabolism genes on OP50 diet over HT115 diet in WT and *str-2 (ok3148)* animals. (G) Lipid droplets stained by Oil Red O (scale bar 50 µM), and (H) Quantification of lipid stores in WT and *str-2* *(ok3148)* animals fed OP50 or HT115 diet. All animals were grown at 25°C irrespective of the diet. *=p < 0.05, **=p < 0.01, *** = p < 0.001, ns=non significant. See also Table S2.

**Figure S8: Life span of wild type *C. elegans* at different temperatures.** Kaplan-Meier survival curves of WT animals at 15°C, 20°C and 25°C fed on *E. coli* OP50. See Table S1 for lifespan conditions and statistics.

**Figure S1.**

**Figure S2**

**Figure S3.**

**(A) (B)**

**Figure S4.**

**Figure S5.**


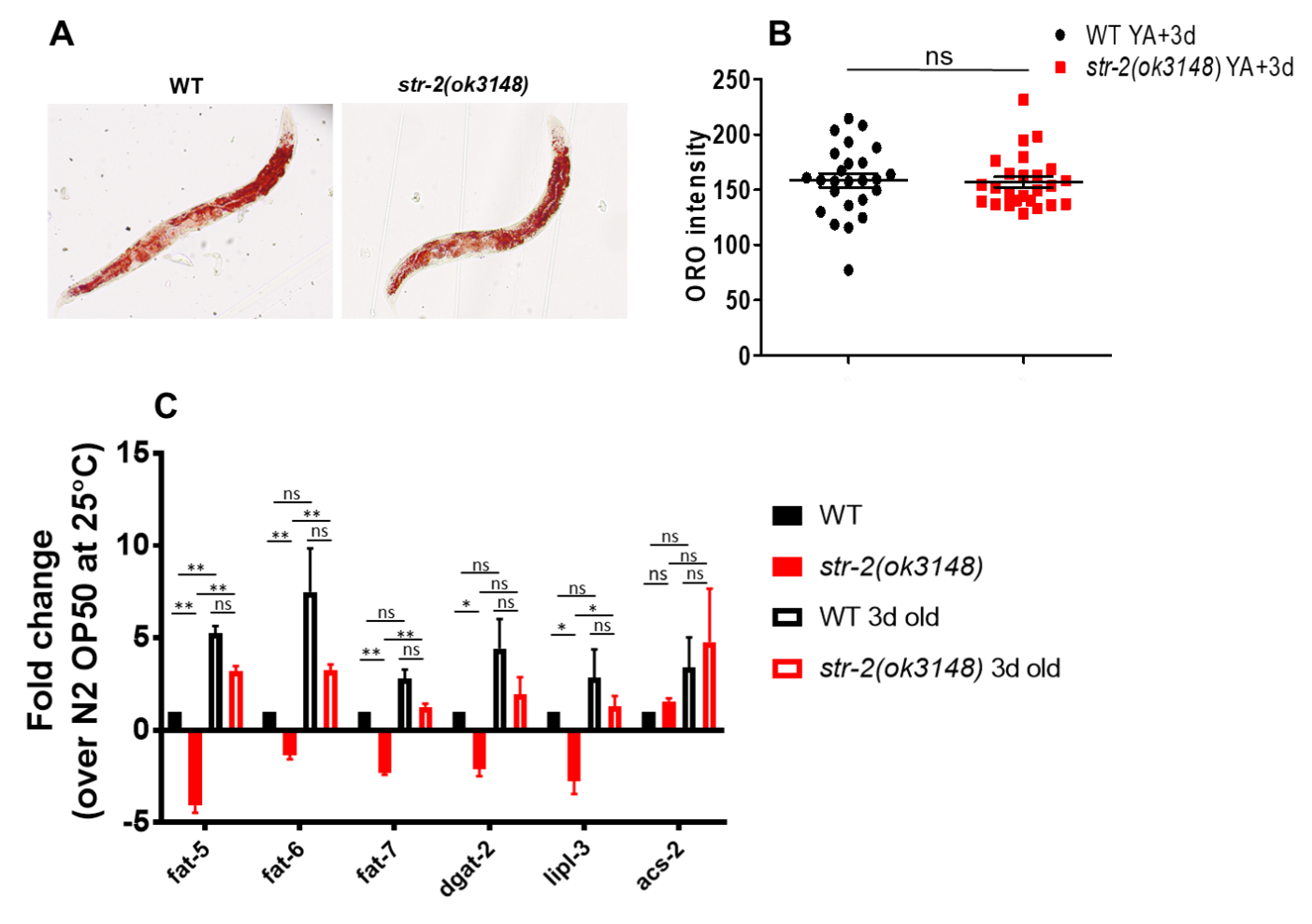


**Figure S6**

**
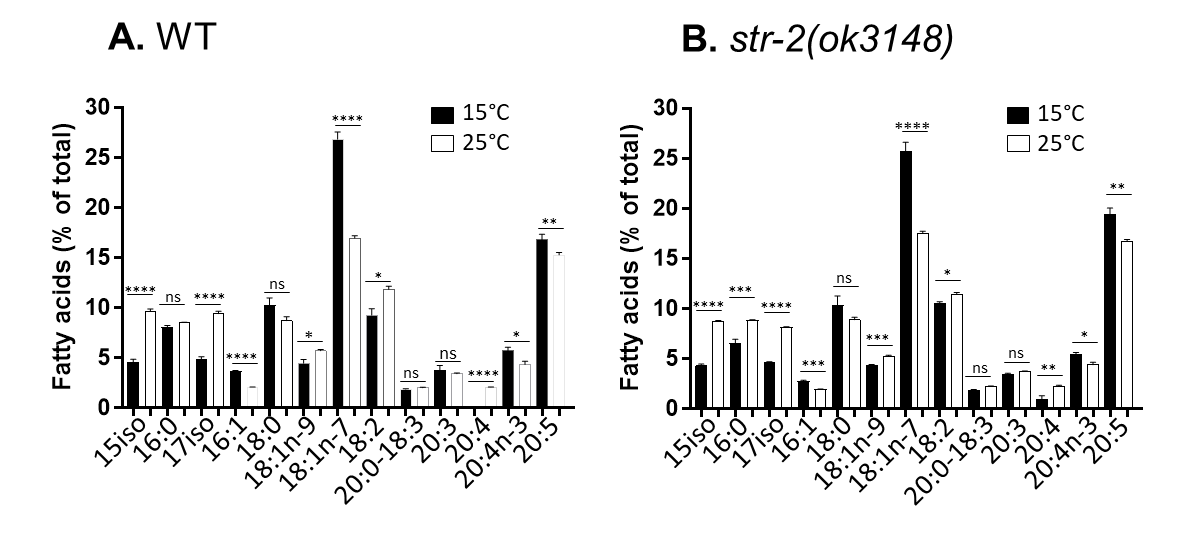
**

**Figure S7.**


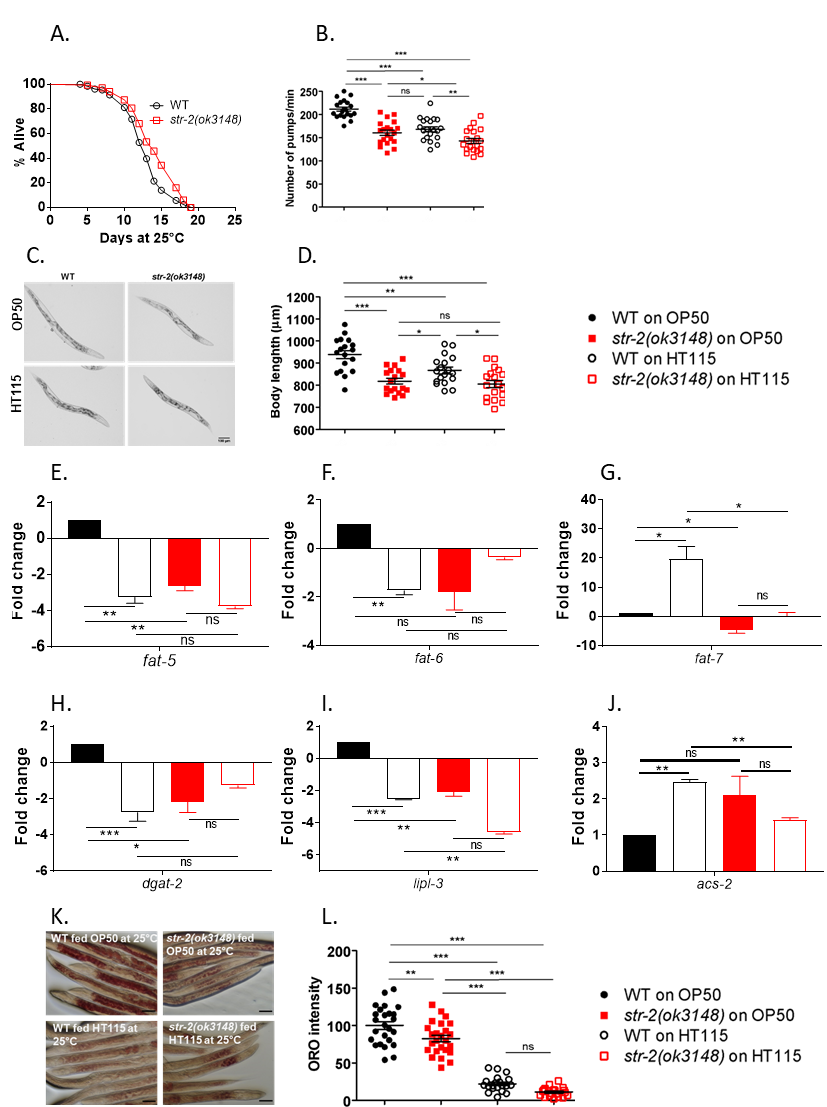


**Figure S8.**
